# The global development trend of occupational therapy applied in stroke: A bibliometric study and visualized analysis via VOSviewer and CiteSpace from 2006 to 2025

**DOI:** 10.1097/MD.0000000000049810

**Published:** 2026-07-17

**Authors:** Long-Xian Liu, Huan-Huan Wang, Ying Dai, Shuang Lu, Guo Yu, Qing-Hua Lai, Meng Wu

**Affiliations:** a Nursing Department, Shenzhen Hospital (Futian) of Guangzhou University of Chinese Medicine, Shenzhen, Guangdong, China; b The Sixth Clinical School of Guangzhou University of Chinese Medicine, Guangzhou, Guangdong, China.

**Keywords:** bibliometrics, CiteSpace, occupational therapy, stroke, visualization analysis, VOSviewer

## Abstract

**Background:**

Occupational therapy (OT) is an important component of stroke rehabilitation, but the global research landscape, collaboration patterns, research hotspots, and emerging trends in OT for stroke have not been systematically mapped. This bibliometric study aimed to analyze publications on OT for stroke from 2006 to 2025 and provide evidence for future clinical practice and research planning.

**Methods:**

English-language articles related to OT for stroke were retrieved from the Web of Science Core Collection from January 1, 2006, to October 31, 2025. After applying predefined inclusion and exclusion criteria, bibliometric and visualization analyses were performed using VOSviewer and CiteSpace 6.4.R1. Publication trends, countries, institutions, authors, journals, keyword co-occurrence, keyword clustering, timeline evolution, and burst terms were analyzed.

**Results:**

A total of 770 publications were included. The annual number of publications showed a fluctuating but overall upward trend, with the highest output observed in 2021 and 2022, each with 60 publications. The United States, Australia, the United Kingdom, and China were the leading contributing countries, while Jikei University and La Trobe University were the most productive institutions. The most prolific authors were Masahiro Abo and Louise Gustafsson. The *British Journal of Occupational Therapy* and *Topics in Stroke Rehabilitation* were the leading journals in terms of publication output. Keyword analyses showed that early research mainly focused on upper-limb function, functional recovery, and basic rehabilitation interventions, whereas recent hotspots have expanded to neurorehabilitation, knowledge translation, virtual reality, clinical trials, mental health, and home-based rehabilitation.

**Conclusion:**

Research on OT for stroke has increased overall over the past 2 decades, with evolving hotspots from impairment-oriented rehabilitation toward broader, occupation-centered, participation-oriented, technology-assisted, and home-based rehabilitation approaches. However, collaboration among countries, institutions, and authors remains relatively fragmented. Future studies should strengthen international and interdisciplinary cooperation and generate high-quality clinical evidence to support OT practice in stroke rehabilitation.

## 1. Introduction

Stroke, including ischemic stroke, intracerebral hemorrhage, and subarachnoid hemorrhage, is a major cause of long-term disability and socioeconomic burden worldwide.^[1,2]^ Poststroke impairments commonly affect motor function, cognition, mood, and activities of daily living, thereby limiting patients’ independence and social participation.^[3,4]^ Occupational therapy (OT) is an essential component of stroke rehabilitation because it aims to improve patients’ ability to participate in meaningful daily activities through functional training, environmental adaptation, assistive strategies, and psychosocial support.^[5–8]^

With the development of stroke rehabilitation, research on OT has expanded from conventional upper-limb and activities-of-daily-living training to broader topics such as cognitive rehabilitation, technology-assisted interventions, community reintegration, and home-based rehabilitation.^[6–8]^ However, the global knowledge structure, collaboration patterns, major contributors, research hotspots, and emerging trends in OT for stroke have not been systematically mapped. A bibliometric analysis may help researchers and clinicians understand how this field has evolved, identify influential countries, institutions, authors, and journals, and clarify future research directions.^[9]^ Therefore, this study aimed to analyze publications on OT for stroke from 2006 to 2025 using CiteSpace and VOSviewer. Specifically, we sought to describe annual publication trends, identify collaboration networks among countries, institutions, and authors, examine journal distribution, and reveal keyword co-occurrence patterns, research hotspots, and emerging frontiers in this field.

## 2. Methods

### 2.1. Literature search strategy

The literature search for this study was conducted using the Web of Science Core Collection (WoSCC) database, which applies selective journal coverage and provides a widely used bibliometric dataset. This approach helped improve the consistency and reliability of the retrieved literature.^[10]^ Document types were limited to research articles, with a focus on English-language publications. The time frame encompassed the period from January 1, 2006, to October 31, 2025. The starting year (2006) was selected because Web of Science records on OT for stroke were sparse before this period and began to show stable indexing and publication growth from 2006 onward. The search strategy expression is detailed in Table 1. Although “cerebral apoplexy” is not commonly used in contemporary scientific terminology, it was included only as an additional search term to improve retrieval sensitivity. Because “OT” may have multiple meanings, records retrieved using “cerebral apoplexy” or “OT” were manually screened by title and abstract to confirm that they were explicitly related to OT in the context of stroke or poststroke rehabilitation.

**Table 1:** Literature search strategy.

Step	Literature search strategy	Articles
#1	(TS = (Occupational Therapy)) OR TS = (“OT”)	31,629
#2	(((TS = (stroke)) OR TS = (cerebral apoplexy)) OR TS = (Stroke rehabilitation)) OR TS = (after stroke)	445,243
#3	(#1) AND #2	2132
#4	(#1) AND #2 and English (Languages) and Article (Document Types) and 2025 or 2024 or 2023 or 2022 or 2021 or 2020 or 2019 or 2018 or 2017 or 2016 or 2015 or 2014 or 2013 or 2012 or 2011 or 2010 or 2009 or 2008 or 2007 or 2006 (Publication Years)	1571

TS = topic search.

### 2.2. Inclusion and exclusion criteria of literature

The literature selection for this study was conducted based on the following criteria: studies were included if they explicitly addressed the application of OT in stroke rehabilitation, and stroke-related publications were considered eligible when they focused on stroke in general or on specific stroke types, including ischemic stroke, intracerebral hemorrhage, subarachnoid hemorrhage, or cerebrovascular accident, provided that the study context was OT or poststroke rehabilitation; eligible publication types were limited to research articles related to OT in stroke rehabilitation. Exclusion criteria were as follows: nonacademic literature, such as conference papers, technical reports, news articles, and commercial advertisements; publications with missing data, incomplete key information, or duplicate literature; studies for which the full text was inaccessible; and non-research literature, including books, newspapers, editorials, monographs, correspondence, news reports, and conference abstracts.

The literature screening process was conducted independently by 2 researchers (Long-Xian Liu and Huan-Huan Wang). All retrieved records were further screened manually by title and abstract to confirm that the included studies were explicitly related to stroke or poststroke rehabilitation. In cases of disagreement, a third senior researcher was consulted to facilitate discussion until consensus was reached.

### 2.3. Data analysis and visualization

**Figure 1.**
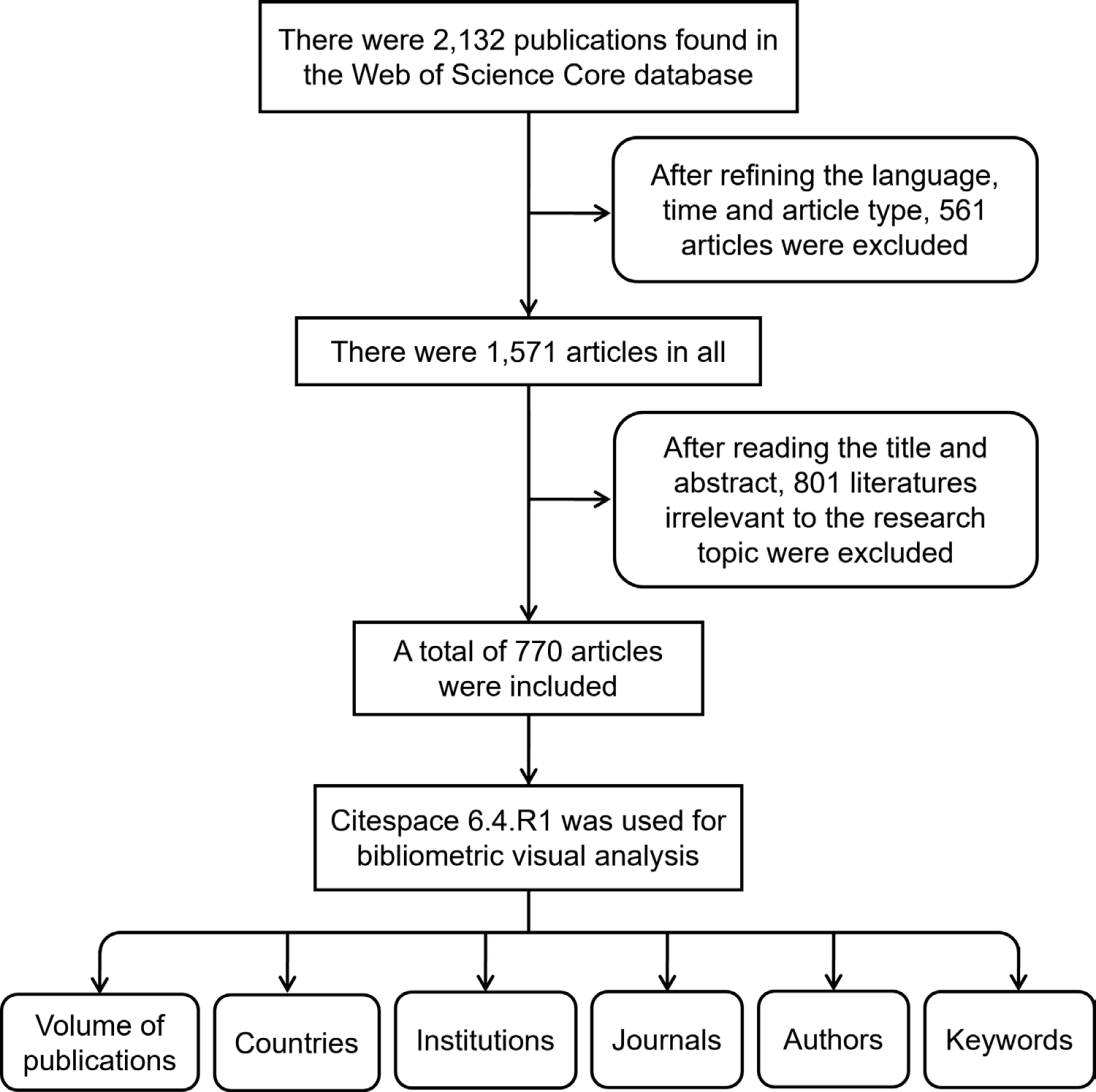
Bibliometric analysis flowchart.

This study utilized a bibliometric method, with literature data retrieved and exported in TXT format. Visualization analysis was conducted using VOSviewer (Centre for Science and Technology Studies, Leiden University) and CiteSpace 6.4.R1 software (Chaomei Chen, Drexel University), with the time span configured from 2006 to 2025, the time slice set to 1 year, and the Top N value defined as 50. The analytical procedure is illustrated in Figure 1. For network pruning, the Pathfinder algorithm was employed, and pruning was executed on each time-slice network while all other parameters remained at their system defaults. The visualization analysis encompassed several dimensions, including the number of publications, authors, countries, research institutions, and literature co-citation. Additionally, keywords served as the analytical unit for bibliometric analyses, which included co-occurrence analysis, cluster analysis, and burst term detection. This study adhered to the Preliminary Guidelines for Bibliometric Review Reports of Biomedical Literature.^[11]^ Because this study was based on published bibliographic metadata rather than full-text clinical evaluation, no formal quality assessment of individual studies was performed. The search strategy was designed to ensure the reproducibility and transparency of bibliometric data retrieval.

### 2.4. Data cleaning

Data cleaning was conducted, including the removal of redundant data, the elimination of duplicate records, and the standardization of spellings for author names, institution names, and keywords. This process included the following scenarios: different spellings or formats of the same country name, such as merging “USA” and “United States of America” into “USA,” and, when calculating national publication output, standardizing regional labels such as England, Scotland, and Wales as the United Kingdom; variations or abbreviations of the same author’s name, which were resolved using Open Researcher and Contributor ID information and author affiliations.

### 2.5. Ethical statement

Ethical approval was not required for this study because it was based exclusively on publicly available bibliographic data and did not involve human participants, animal experiments, or identifiable personal information. Therefore, informed consent was not applicable.

## 3. Results

### 3.1. Annual number and trend of articles

**Figure 2.**
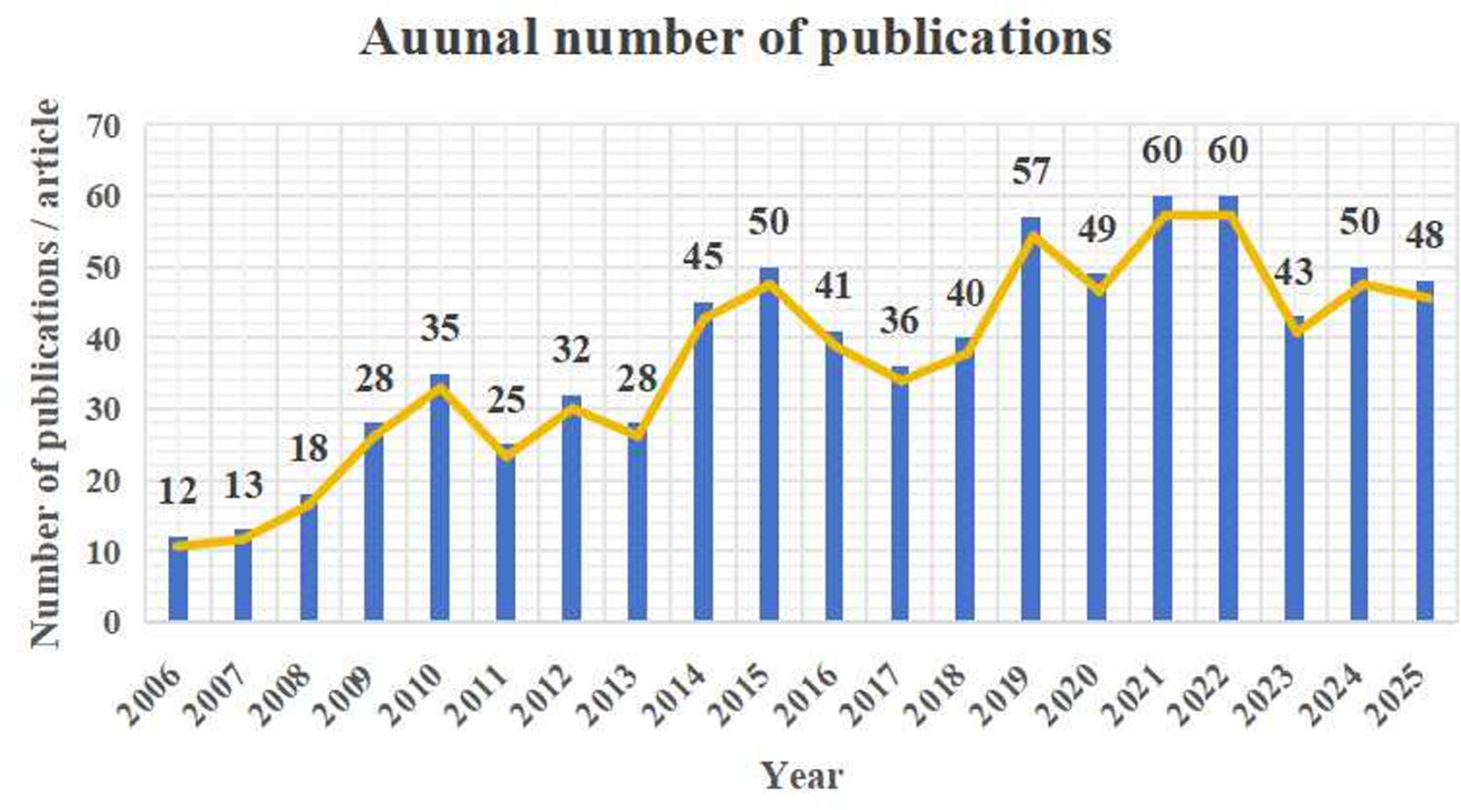
Graph of the growth trend in the number of publications.

A total of 2132 literature entries were initially identified. After applying publication type, language, time span, and title/abstract screening criteria, 770 papers focusing on the application of OT in stroke were ultimately included. The number of publications exhibited an overall fluctuating upward trend. From 2006 to 2015, the number of publications showed a pattern of slow fluctuation. Following a 2-year decline after 2015, a slight fluctuating growth resumed, culminating in peak values in both 2021 and 2022. A minor decrease was observed in 2023, after which an upward trajectory reemerged. Because the search ended on October 31, 2025, the publication count for 2025 should be interpreted as a partial-year estimate (see Fig. 2).

### 3.2. Distribution of countries

**Figure 3.**
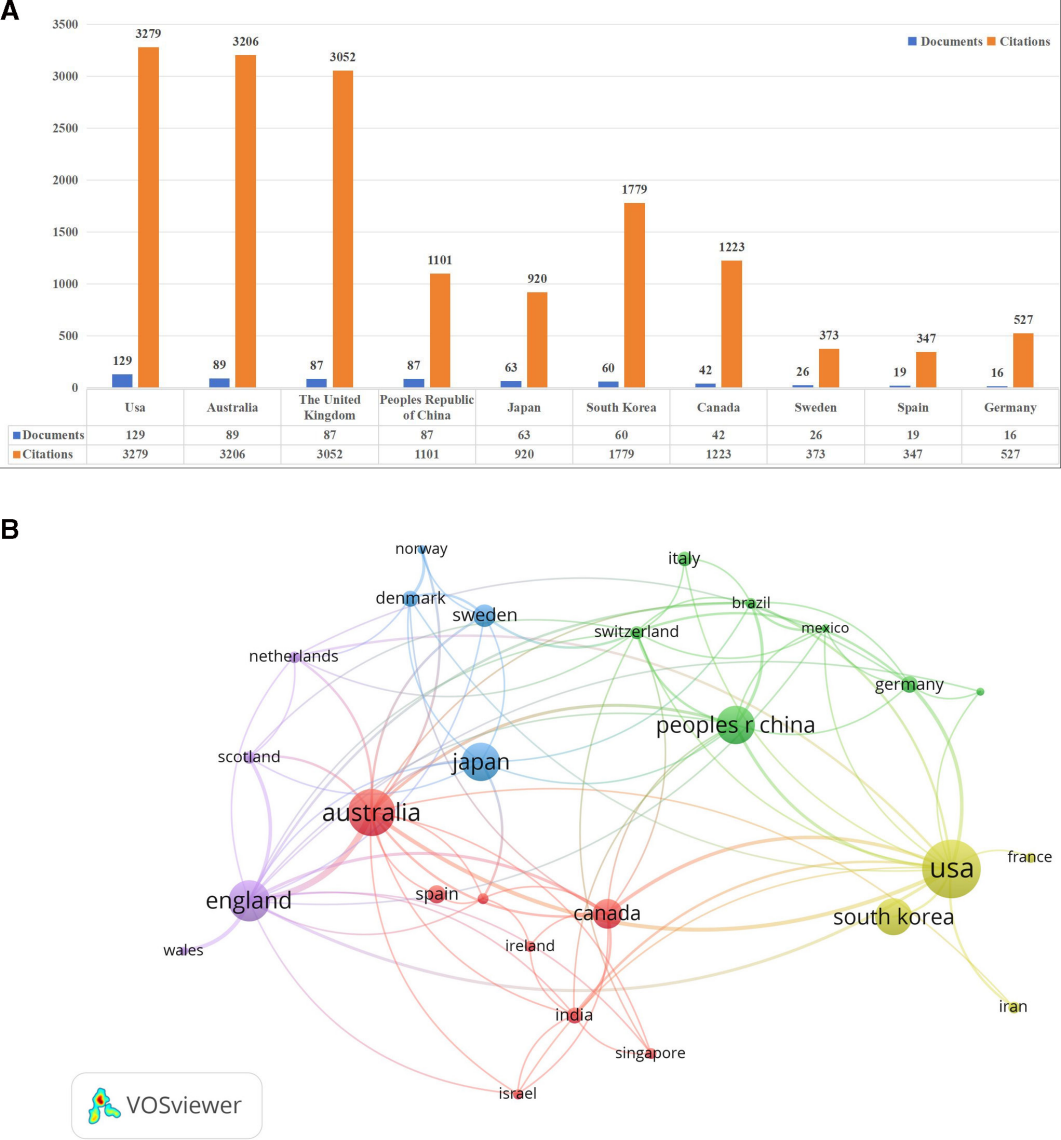
Country/region distribution and collaboration network of occupational therapy research for stroke. (A) The number of published papers and citation counts of the top 10 countries in terms of the number of research papers on the application of occupational therapy in the field of stroke. (B) Graph of country/region co-occurrence analysis of occupational therapy applied in the field of stroke.

Figure 3A presents the academic output of the top 10 countries or regions in this field, ranked by the number of publications. The United States leads with 129 publications, followed by Australia with 89, while both the United Kingdom and China contributed 87 publications each. Japan and South Korea also produced more than 50 publications, while Canada contributed 42 publications. Notably, among these productive publishing entities, only China is classified as a developing country, whereas the others are developed nations. Research from the United States not only leads in volume but also in academic impact, accumulating 3279 citations, followed by Australia and the United Kingdom with 3206 and 3052 citations, respectively. The geographical distribution of the included studies was analyzed using VOSviewer, resulting in a co-occurrence network as illustrated in Figure 3B. This network is characterized by 5 major country/region-related clusters, mainly involving the United States, Australia, the United Kingdom, China, and Japan. Among these clusters, the strongest collaborative link is observed between Australia and the United Kingdom, highlighting a key axis of academic cooperation in this research domain.

### 3.3. Analysis of institutions

**Figure 4.**
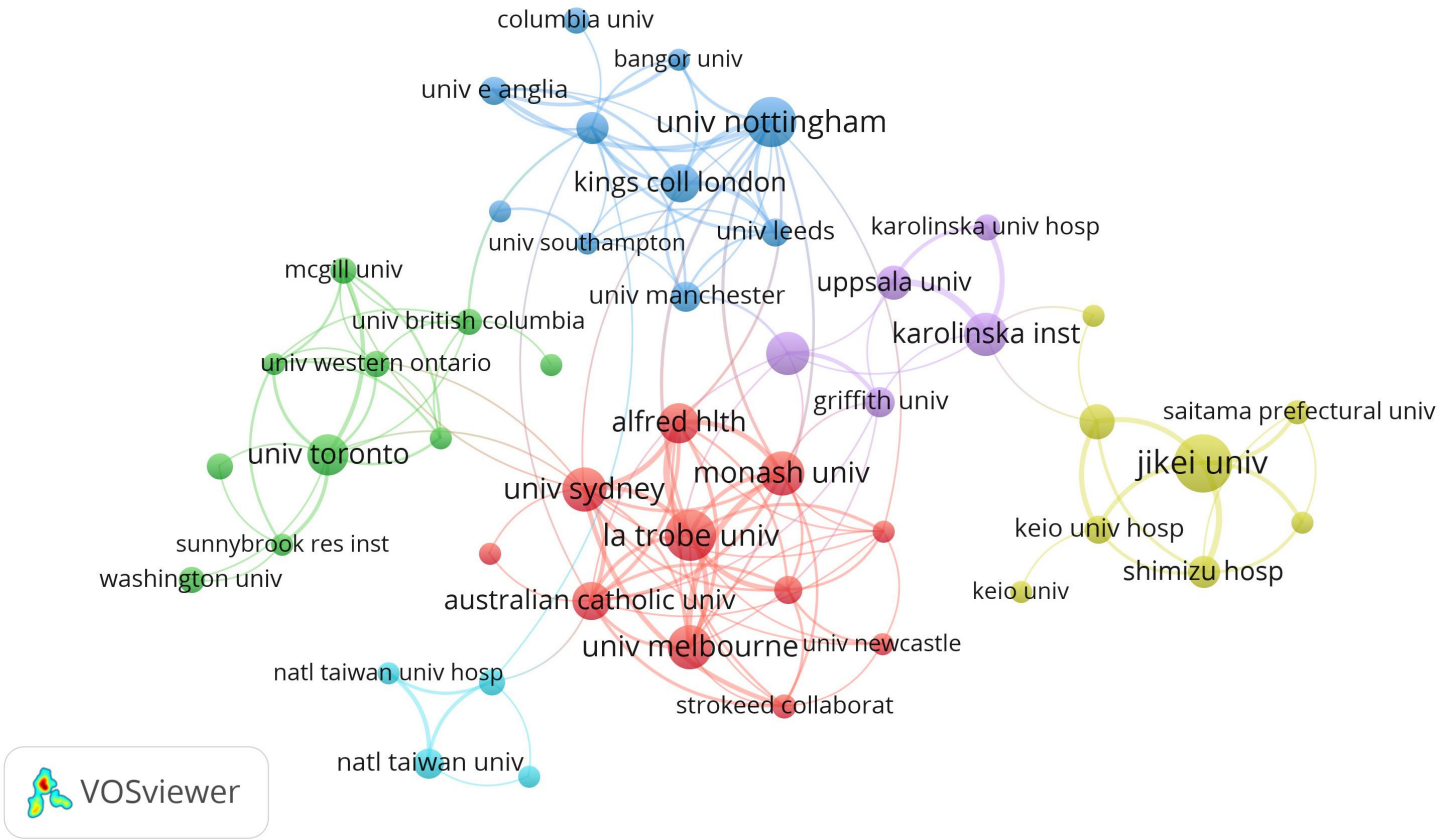
Institutional co-occurrence network of occupational therapy research for stroke.

The institutional analysis identified 48 institutions that met the visualization threshold in the dataset of 770 articles. Table 2 displays the top 10 institutions by publication output, with Jikei University in Japan leading with 30 papers, the highest number of publications. Following closely are La Trobe University in Australia with 24 publications and the University of Nottingham in the United Kingdom with 23 papers. La Trobe University also stands out with the highest citation count, amassing 2358 citations. In Figure 4, the inter-institutional cooperation network among key contributors in the field of stroke OT is depicted. This network highlights 5 key institutions – Jikei University, La Trobe University, the University of Nottingham, Karolinska Institutet, and the University of Toronto – forming distinct cooperative networks. These institutions formed several visible cooperative clusters; however, the connections between some clusters remained relatively limited, suggesting that inter-institutional collaboration could be further strengthened.

**Table 2:** The top 10 productive institutions regarding the research on occupational therapy applied in the field of stroke.

Rank	Institution	Country	Count	Citations
1	Jikei University	Japan	30	512
2	La Trobe University	Australia	24	2358
3	University of Nottingham	United Kingdom	23	297
4	University of Sydney	Australia	18	428
5	Monash University	Australia	18	225
6	University of Melbourne	Australia	18	240
7	University of Queensland	Australia	17	288
8	Karolinska Institutet	Sweden	17	301
9	University of Toronto	Canada	16	466
10	Alfred Health	Australia	15	354

### 3.4. Analysis of journals

**Figure 5.**
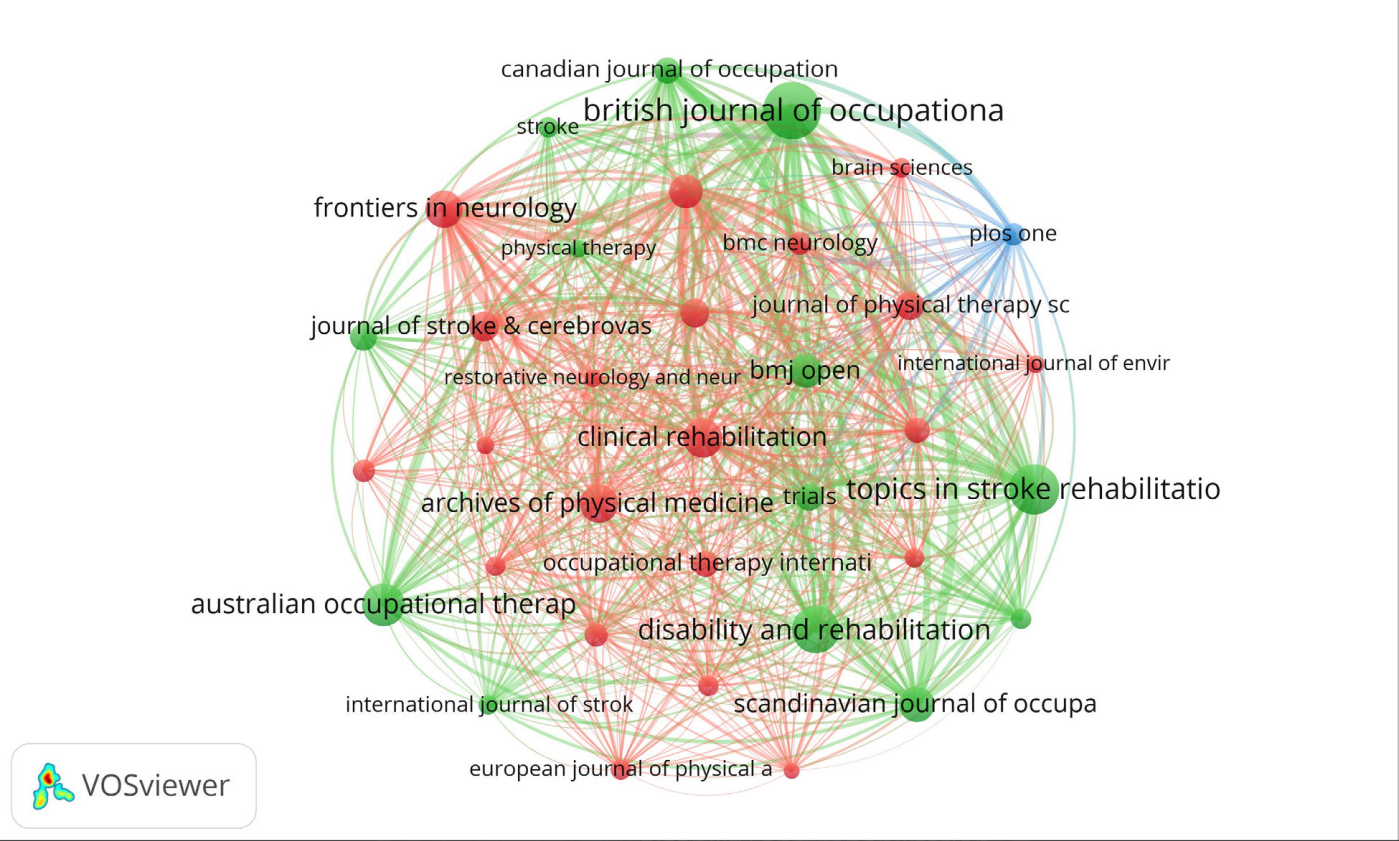
Journal co-citation network of occupational therapy research for stroke.

In the journal analysis, 35 journals met the visualization threshold. Table 3 shows that the top 10 journals published 282 articles, accounting for 36.6% of all included publications. The *British Journal of Occupational Therapy* had the highest number of publications, with 50 articles, followed by *Topics in Stroke Rehabilitation* with 38 and *Disability and Rehabilitation* with 35. The *Journal of Neuroengineering and Rehabilitation* had the highest impact factor (IF), at 5.2. Six journals had IFs ranging from 2 to 5, including *Topics in Stroke Rehabilitation*, *Disability and Rehabilitation*, *Clinical Rehabilitation*, *Archives of Physical Medicine and Rehabilitation*, *Frontiers in Neurology*, and *BMJ Open*. Three journals, namely the *British Journal of Occupational Therapy*, *Australian Occupational Therapy Journal*, and *Scandinavian Journal of Occupational Therapy*, had IFs below 2. Journal co-citation analysis was used to identify journals that formed the knowledge base and citation relationships in this field. Co-citation analysis was performed using VOSviewer, as shown in Figure 5. The node size indicates the co-citation frequency of each journal, while the connecting lines represent co-citation relationships among journals. In the color-coded cluster analysis, the red cluster represents journals focusing on the integration of rehabilitation medicine and OT, such as the *British Journal of Occupational Therapy*, *Topics in Stroke Rehabilitation*, and *Disability and Rehabilitation*. The green cluster represents journals focusing on the convergence of rehabilitation medicine and neurology, including prominent publications such as *Clinical Rehabilitation*, *Archives of Physical Medicine and Rehabilitation*, and *Frontiers in Neurology*. Journals of a general nature, such as *PLOS One*, are categorized within the blue cluster.

**Table 3:** The top 10 journals that published articles regarding the research on occupational therapy applied in the field of stroke.

Rank	Journal title	Country	Count	Citations	JCR	IF
1	*British Journal of Occupational Therapy*	United Kingdom	50	291	Q4	0.9
2	*Topics in Stroke Rehabilitation*	United States	38	653	Q1	2.5
3	*Disability and Rehabilitation*	United Kingdom	35	493	Q2	2
4	*Australian Occupational Therapy Journal*	Australia	29	340	Q2	1.8
5	*Clinical Rehabilitation*	United Kingdom	25	523	Q1	2.9
6	*Archives of Physical Medicine and Rehabilitation*	United States	24	735	Q1	3.7
7	*Frontiers in Neurology*	Switzerland	22	233	Q2	2.8
8	*Scandinavian Journal of Occupational Therapy*	Sweden	21	360	Q3	1.3
9	*BMJ Open*	United Kingdom	19	135	Q2	2.3
10	*Journal of Neuroengineering and Rehabilitation*	United Kingdom	19	810	Q1	5.2

IF = impact factor, JCR = Journal Citation Reports, Q = quartile.

### 3.5. Analysis of authors

**Figure 6.**
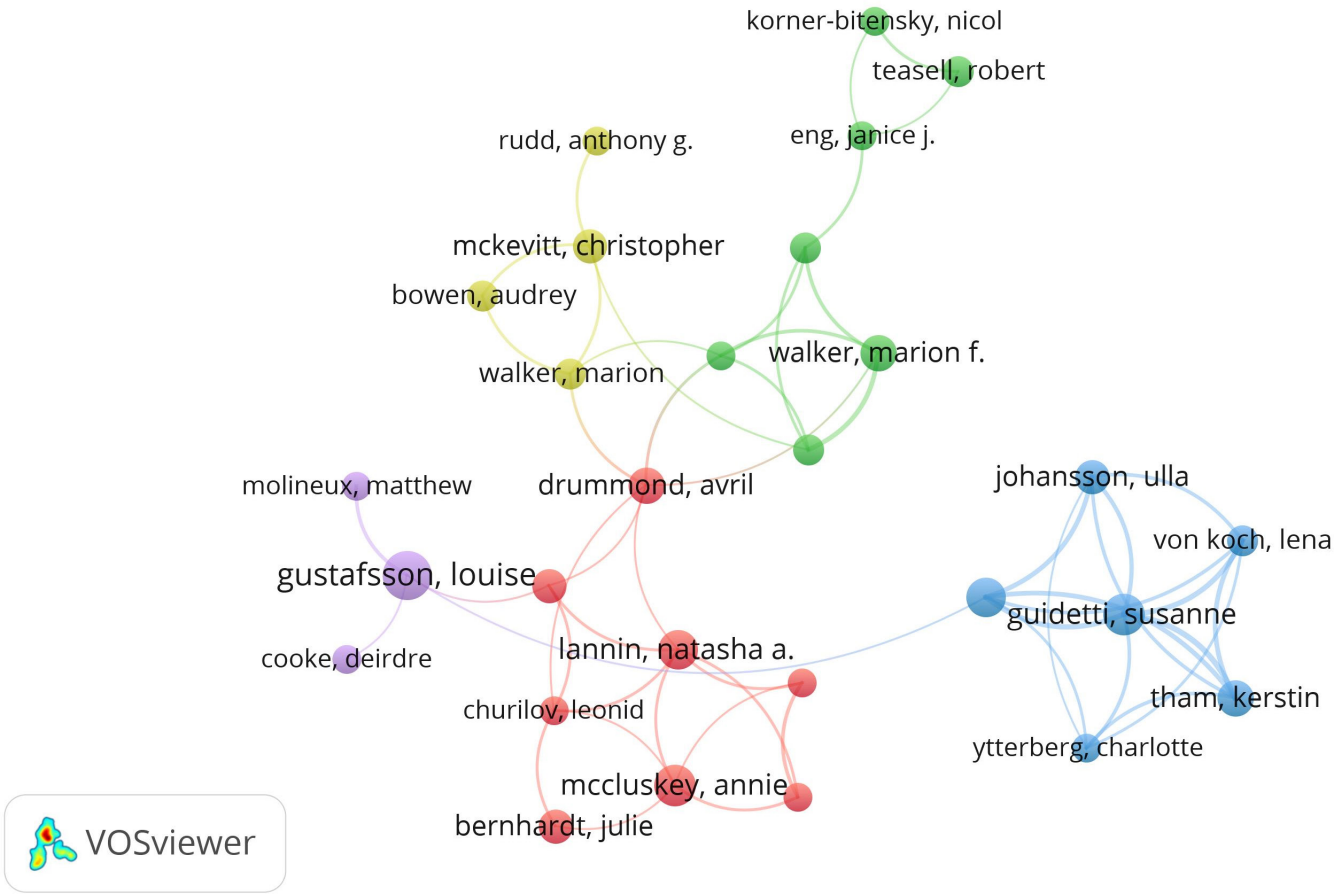
Author co-occurrence network of occupational therapy research for stroke.

A total of 5319 authors participated in research on the utilization of OT in stroke literature. The top 10 most prolific authors are detailed in Table 4. Masahiro Abo from Jikei University in Japan stands out as the most productive author, having published 30 articles and receiving the highest number of citations (492). The majority of these leading authors are affiliated with academic institutions in Japan, Sweden, and Australia. The author co-occurrence analysis, visualized in Figure 6 using VOSviewer, reveals distinct collaborative subnetworks denoted by clusters of various colors. For instance, the green cluster is centered on Marion F. Walker, while the red cluster is formed around Annie McCluskey. Collaboration appeared relatively strong within some clusters, but connections between different author groups remained limited.

**Table 4:** The top 10 productive authors regarding research on occupational therapy applied in the field of stroke.

Rank	Author	Institution	Country	Count	Citations
1	Abo, Masahiro	Jikei University	Japan	30	492
2	Gustafsson, Louise	University of Queensland	Australia	16	121
3	Kakuda, Wataru	Jikei University	Japan	13	355
4	Yamada, Naoki	Jikei University	Japan	11	131
5	Guidetti, Susanne	Karolinska Institutet	Sweden	11	232
6	McCluskey, Annie	University of Sydney	Australia	11	84
7	Eriksson, Gunilla	Karolinska Institutet	Sweden	10	191
8	Lannin, Natasha A.	Monash University	Australia	10	252
9	Tham, Kerstin	Karolinska Institutet	Sweden	8	199
10	Senoo, Atsushi	Tokyo Metropolitan University	Japan	8	67

### 3.6. Analysis of keywords

**Figure 7.**
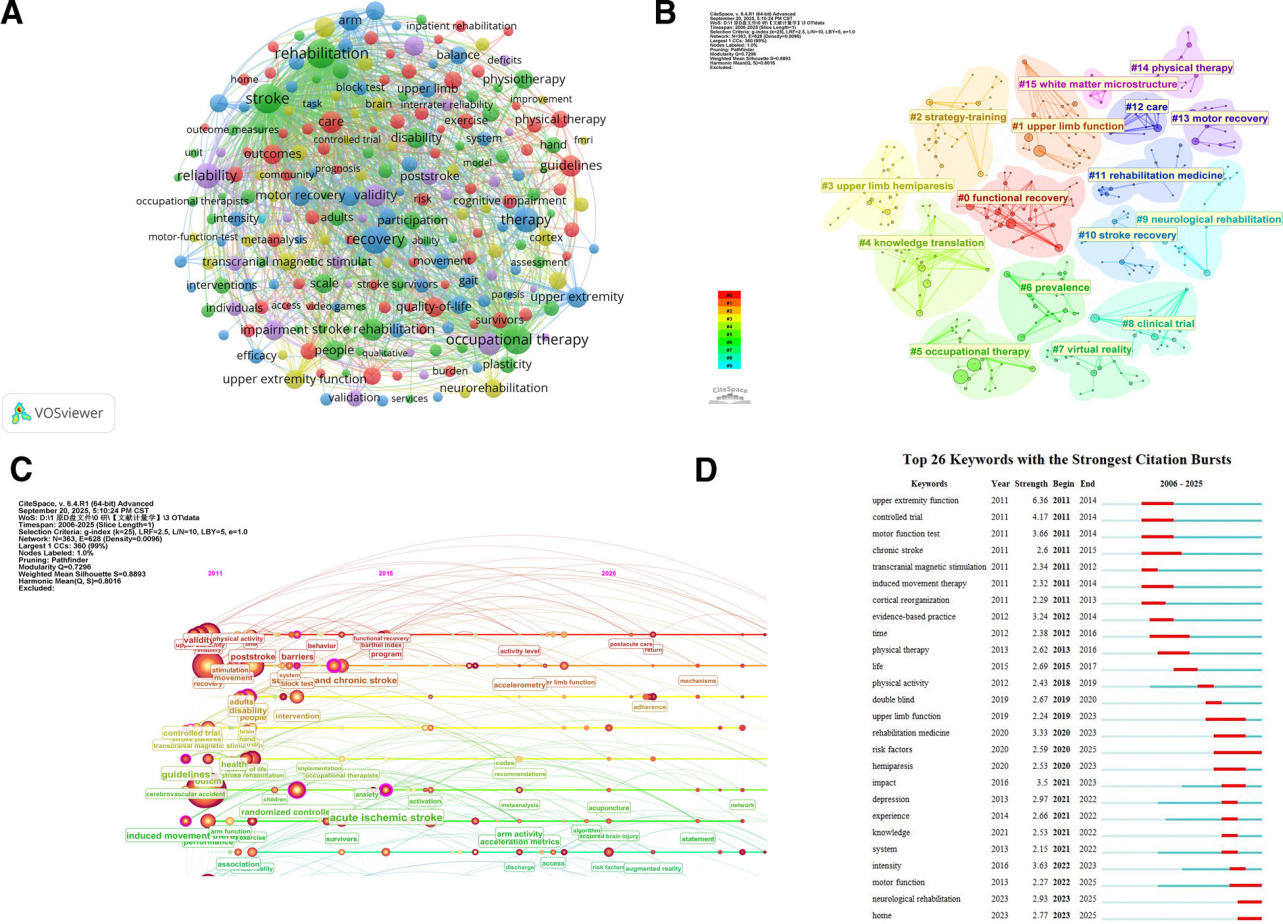
Keyword co-occurrence, clustering, timeline, and burst analyses of occupational therapy research for stroke. (A) Keyword co-occurrence network of occupational therapy research for stroke. (B) Keyword clustering network of occupational therapy research for stroke. (C) Keyword timeline map of occupational therapy research for stroke. (D) Keyword burst analysis of occupational therapy research for stroke.

Keywords hold significant importance in research articles, with high-frequency or burst keywords providing insights into current research trends. Table 5 lists the top 20 high-frequency keywords. Among them, the 5 most frequent keywords were occupational therapy, rehabilitation, recovery, reliability, and stroke. Figure 7A illustrates a keyword co-occurrence network created using VOSviewer.

**Table 5:** Top 20 high-frequency keywords in the field of occupational therapy applied to stroke.

No.	Keywords	Year of initial appearance	Frequency	Centrality
1	occupational therapy	2011	209	0
2	rehabilitation	2011	181	0.02
3	recovery	2011	144	0.05
4	reliability	2011	104	0.04
5	stroke	2011	77	0.05
6	therapy	2012	74	0.02
7	upper extremity	2011	68	0.03
8	upper limb	2011	67	0.1
9	validity	2011	62	0.08
10	stroke rehabilitation	2012	61	0.03
11	care	2011	59	0.05
12	motor recovery	2014	51	0.12
13	performance	2011	50	0.05
14	quality of life	2012	44	0.06
15	outcome	2011	44	0.05
16	impairment	2011	43	0.01
17	activities of daily	2012	42	0.07
18	upper extremity function	2011	40	0.05
19	Arm	2011	40	0.07
20	Scale	2011	39	0.03

Based on keyword characteristics, the terms were organized into 16 clusters. The keyword clustering diagram generated using CiteSpace is presented in Figure 7B, with corresponding cluster labels listed in Table 6. Utilizing the log-likelihood ratio algorithm, a modularity value (*Q*) = 0.7296 and a silhouette value (*S*) = 0.8893 were achieved, indicating that the clustering results are both significant and robust.^[12]^ Clusters #0 (functional recovery), #10 (stroke recovery), and #13 (motor recovery) are primarily associated with the trajectories of functional recovery in poststroke patients. Clusters #1 (upper limb function) and #3 (upper limb hemiparesis) predominantly reflect the most common upper-limb dysfunctions following stroke. Clusters #2 (strategy-training), #5 (occupational therapy), #8 (clinical trial), #9 (neurological rehabilitation), #11 (rehabilitation medicine), and #14 (physical therapy) are identified as representing specific intervention approaches within OT, along with their intersections with related disciplines. Other clusters – including #4 (knowledge translation), #6 (prevalence), #7 (virtual reality), #12 (care), and #15 (white matter microstructure) – illustrate the broad scope of OT research in stroke, encompassing areas such as knowledge translation, technology application, and underlying mechanisms.

**Table 6:** Keyword cluster label information of occupational therapy applied in the field of stroke.

Cluster ID	Size	Silhouette	Year	Label (LLR)
#0	42	0.957	2015	functional recovery; vocational rehabilitation; upper extremity; disability; lifestyle
#1	33	0.893	2017	upper limb function; subacute stroke; spatial attention; virtual reality; fugl meyer assessment
#2	31	0.847	2017	strategy-training; community; occupational performance; assistive devices; music
#3	28	0.86	2014	upper limb hemiparesis; transcranial magnetic stimulation; repetitive transcranial magnetic stimulation; cortical reorganization; botulinum toxin type a
#4	27	0.948	2014	knowledge translation; stroke rehabilitation; evidence-based practice; health services research; occupational therapists
#5	27	0.868	2017	occupational therapy; cerebrovascular accident; complex intervention; goal setting; physiotherapy
#6	24	0.762	2016	prevalence; validation; impact; exercise prescription; mortality
#7	24	0.86	2019	virtual reality; augmented reality; speech therapy; organized stroke care; pathway
#8	22	0.872	2015	clinical trial; robotics; acute stroke; motor function; activities of daily living
#9	21	0.946	2019	neurological rehabilitation; ischemic stroke; activity; 1st year; creative activities
#10	19	0.891	2016	stroke recovery; transcranial direct current stimulation; neuroscience; tactile perception; exoskeleton rehabilitation
#11	16	0.788	2018	rehabilitation medicine; stroke medicine; physical therapy modalities; poststroke checklist; apraxia
#12	15	0.895	2012	care; guidelines; prevention; protocols; patient preference
#13	13	0.99	2014	motor recovery; brain stimulation; fMRI; reorganization; upper limb
#14	13	0.954	2016	physical therapy; impairment; chronic pain; hospital costs; clinical audit
#15	5	0.988	2017	white matter microstructure; non-fluent aphasia; LOTCA; TBSS; PSA

fMRI = functional magnetic resonance imaging, LLR = log-likelihood ratio, LOTCA = Loewenstein Occupational Therapy Cognitive Assessment, PSA = post-stroke aphasia, TBSS = tract-based spatial statistics.

The timeline graph effectively illustrates the evolving pathways of research hotspots as indicated by keywords. Within specific research themes, it also aids in revealing fluctuations in term popularity and the temporal patterns associated with keyword clustering.^[13]^ The keyword timeline graph generated by CiteSpace is depicted in Figure 7C. An analysis of the keyword timeline graph from the WoSCC indicated that OT in stroke rehabilitation has been consistently examined by researchers worldwide throughout the study period. The curves in the graph reflect the connectivity strength among keyword nodes. Initially, the field was predominantly influenced by #0 (functional recovery). By 2025, clusters such as #0 (functional recovery), #1 (upper limb function), #2 (strategy-training, #4 (knowledge translation), #5 (occupational therapy), #7 (virtual reality), #8 (clinical trial), and #9 (neurological rehabilitation) remained active and continued to advance the research domain.

Keyword burst analysis is a valuable approach for identifying research hotspots and emerging trends in particular years, providing insightful views on the development of a specific field. Using CiteSpace, the top 26 keywords with the strongest bursts from 2006 to 2025 were determined, as depicted in Figure 7D. Among these keywords, “upper extremity function” exhibited the strongest burst strength, measuring 6.36. “Motor function,” “neurological rehabilitation,” and “home” emerged as recent burst terms and were anticipated to sustain considerable research attention in the future.

## 4. Discussion

### 4.1. Overview of the results

A total of 770 articles on OT in stroke rehabilitation were analyzed in this study using VOSviewer and CiteSpace to identify research hotspots and trends.^[14–16]^ The annual publication trend showed several periods of fluctuation and growth. Initially, from 2006 to 2015, the utilization of OT in stroke recovery was not prominent.^[17]^ This limited adoption may be related to the early stage of research and the need to evaluate the cost-effectiveness and practical value of OT interventions.^[18]^ Despite the relatively low number of publications during this period, a consistent upward trend was observed. In the 2010s, there were 3 minor peaks in the annual number of publications, occurring in 2010, 2015, and 2019, with 35, 50, and 57 publications, respectively. This trend indicates a growing interest in the field, likely driven by the broader development of technology-assisted rehabilitation approaches, particularly robotics and virtual reality.^[19,20]^ A slight decrease in research on OT for stroke rehabilitation was observed after 2022, although the underlying reasons require further investigation. In recent years, epidemiological studies have reported an increasing incidence of stroke over the past decade, particularly among younger populations,^[21]^ along with growing global attention to rehabilitation service accessibility and integration into health systems.^[22,23]^ Accordingly, poststroke rehabilitation has received increasing research and clinical attention as part of broader strategies to reduce the global burden of stroke.^[24]^

Consequently, there has been a growing emphasis on the use of OT in stroke rehabilitation, which is recognized as an effective intervention for stroke recovery, leading to a rapid expansion of its clinical significance and a surge in research interest.^[15,25]^ By 2020, the cumulative number of publications in this field had exceeded 500, indicating a significant increase in research activity. The peak annual number of publications was reached in 2021 and 2022, with 60 publications each year. It is expected that this research area will continue to attract substantial scholarly attention in the future.

An analysis of global research publication distribution reveals that most countries worldwide have contributed to research on OT in stroke rehabilitation. Notably, among the top 10 countries in publication output, most are developed countries, while China also ranks among the major contributors in this field. The predominance of developed countries may reflect their established rehabilitation systems, stronger research infrastructure, and greater investment in stroke rehabilitation research. China’s contribution may be related to population aging, the increasing burden of stroke, and the growing demand for poststroke rehabilitation services in China.^[2,26,27]^ Because poststroke disability often affects activities of daily living and social participation,^[4]^ OT has received increasing attention as a rehabilitation approach aimed at improving functional independence.^[14,25,28]^ However, international collaboration involving China remains relatively limited, suggesting the need for stronger cross-national and interdisciplinary cooperation in future research.

As shown in Tables 2 and 4 and Figure 6, an examination of researchers and institutions showed that the most active contributors were mainly located in Japan, Australia, and several other countries with relatively active rehabilitation research output. This finding suggests that OT for stroke has developed around several active institutional and author groups, but the collaboration network remains relatively fragmented. Approximately half of the top 10 productive authors were affiliated with Japanese institutions; however, publication output and citation impact were not always consistent across authors and institutions. Regarding journal distribution, the top 10 journals accounted for 282 of the 770 included publications, indicating that studies in this field were distributed across a range of OT, rehabilitation, and neurology-related journals. These findings highlight the need to strengthen cross-institutional,international, and interdisciplinary collaboration and promote high-quality clinical research in OT for stroke. Taken together, the distribution of contributors, journals, and research themes suggests that OT for stroke is increasingly developing toward multidisciplinary rehabilitation and technology-assisted stroke care.

### 4.2. Hotspots analysis

Author co-occurrence analysis identified several influential research groups, including those led by Louise Gustafsson, Susanne Guidetti, and Annie McCluskey. Their work reflects 3 major directions in OT for stroke: assessment and management of upper-limb dysfunction, client-centered activities-of-daily-living interventions, and evidence-based implementation of rehabilitation strategies.^[29–34]^ These themes are consistent with the keyword results, which showed that upper limb function, functional recovery, clinical trials, and knowledge translation were central topics in this field.

The contributions of these research groups suggest that OT for stroke has gradually shifted from impairment-oriented training to occupation-centered and participation-oriented rehabilitation.^[17,31,35–37]^ In addition to improving motor and cognitive function, recent studies have emphasized meaningful activities, patient-centered goal setting, community reintegration, and home-based rehabilitation.^[31,37–43]^ These developments indicate that OT is increasingly integrated into comprehensive stroke rehabilitation models.

### 4.3. Keywords and trend analysis

As illustrated in Figure 7C and D, research themes have evolved over time and can be broadly categorized into 3 stages:

1. The first stage (2011–2015): The early literature primarily concentrated on assessment and intervention techniques for motor function rehabilitation through OT following stroke. Key topics included the assessment of upper limb function, methods for testing motor function, transcranial magnetic stimulation, and constraint-induced movement therapy.^[44,45]^ Researchers also validated the effectiveness of OT in promoting motor function recovery after stroke through randomized controlled trials.
2. The second stage (2016–2020): During this period, intervention studies further examined neuromodulation and rehabilitation approaches for stroke-related hemiparesis,^[46]^ reflecting an expanded research scope from general motor recovery toward more specific poststroke impairments and intervention strategies.
3. The third stage (2021–2025): As OT continued to evolve, this phase emphasized the identification and management of risk factors during stroke rehabilitation while also increasing attention to patients’ mental health. A notable aspect of this stage was the transition of neurorehabilitation settings from hospitals to home environments, investigating the feasibility and effectiveness of home-based rehabilitation models, with the ultimate goal of achieving comprehensive coverage of rehabilitation settings.^[47,48]^

In summary, stroke rehabilitation research has advanced from initial explorations of techniques and methods to a comprehensive and refined discipline that integrates clinical practice, holistic patient health, and the development of disciplinary systems. This evolution illustrates the ongoing adaptation and increasing sophistication of OT technologies in meeting the rehabilitation needs of stroke patients. These advancements signify a shift in OT services for stroke from the “laboratory” to “patients’ daily lives,” effectively connecting academic rigor with clinical relevance. Continued high-quality research in this domain is strongly encouraged. Overall, the evolution of OT in stroke reflects a transition from impairment-focused rehabilitation to multidisciplinary, technology-assisted, and patient-centered care models.

## 5. Strengths and limitations

This study has several strengths. The use of the WoSCC as the data source, together with predefined criteria for publication type, language, and time span, helped ensure the reliability and consistency of the bibliometric dataset. By combining CiteSpace and VOSviewer, this study visualized publication trends, collaboration networks among countries, institutions, and authors, journal distribution, keyword co-occurrence, clustering, and burst terms, thereby providing a multidimensional overview of the field. In addition, the specific focus on OT for stroke allowed this study to generate targeted evidence on global research patterns, major contributors, hotspots, and emerging directions, which may inform future clinical practice and research planning.

This study has several limitations. The literature search was confined to the WoSCC, focusing solely on English-language publications and excluding other databases such as PubMed, Scopus, and Chinese-language literature. This limitation may have led to the omission of relevant studies, potentially hindering a comprehensive representation of global research in this field. Despite attempts to standardize data, such as author and institution names, database incompleteness affected the accuracy and thoroughness of the cooperation network analysis. Moreover, bibliometric analysis relies on quantitative aspects of the available literature and lacks the capacity for a detailed assessment of variations in literature quality or the practical effectiveness of OT interventions in clinical settings. Future research should integrate qualitative analyses with empirical clinical studies to validate the research trends identified in this study.

## 6. Conclusion

This bibliometric study mapped the global research landscape of OT for stroke from 2006 to 2025. The number of publications showed an overall upward trend, and the field has evolved from early attention to upper-limb function and basic rehabilitation interventions toward broader themes such as neurorehabilitation, mental health, home-based rehabilitation, and technology-assisted interventions. Although research activity has increased, collaboration among countries, institutions, and authors remains fragmented. Future studies should strengthen international and interdisciplinary cooperation, develop high-quality clinical evidence, and further explore patient-centered and home-based OT strategies for stroke rehabilitation.

## Author contributions

**Formal analysis:** Long-Xian Liu, Huan-Huan Wang, Ying Dai, Shuang Lu, Guo Yu, Meng Wu.

**Data curation:** Huan-Huan Wang, Shuang Lu, Qing-hua Lai.

**Supervision:** Meng Wu.

**Validation:** Meng Wu.

**Writing – original draft:** Long-Xian Liu, Huan-Huan Wang, Ying Dai.

**Writing – review & editing:** Long-Xian Liu, Ying Dai, Guo Yu, Qing-hua Lai, Meng Wu.
